# Does preoperative abduction value affect functional outcome of combined muscle transfer and release procedures in obstetrical palsy patients with shoulder involvement?

**DOI:** 10.1186/1471-2474-5-25

**Published:** 2004-08-03

**Authors:** Atakan Aydin, Turker Ozkan, Defne Onel

**Affiliations:** 1 Department of Plastic and Reconstructive Surgery, Istanbul University, Istanbul Faculty of Medicine, Istanbul, Turkey

## Abstract

**Background:**

Obstetric palsy is the injury of the brachial plexus during delivery. Although many infants with plexopathy recover with minor or no residual functional deficits, some children don't regain sufficient limb function because of functional limitations, bony deformities and joint contractures. Shoulder is the most frequently affected joint with internal rotation contracture causing limitation of abduction, external rotation. The treatment comprises muscle release procedures such as posterior subscapularis sliding or anterior subscapularis tendon lengtening and muscle transfers to restore the missing external rotation and abduction function.

**Methods:**

We evaluated whether the preoperative abduction degree affects functional outcome. Between 1998 and 2002, 46 children were operated on to restore shoulder abduction and external rotation. The average age at surgery was 7.6 years and average follow up was 40.8 months. We compared the postoperative results of the patients who had preoperative abduction less than 90° (Group I: n = 37) with the patients who had preoperative abduction greater than 90° (Group II: n = 9), in terms of abduction and external rotation function with angle measurements and Mallet classification. We inquired whether patients in Group I needed another muscle transfer along with latissimus dorsi and teres major transfers.

**Results:**

In Group I the average abduction improved from 62.5° to 131.4° (a 68.9° ± 22.9°gain) and the average external rotation improved from 21.4° to 82.6° (a 61.1° ± 23°gain). In Group II the average abduction improved from 99.4°to 140°(a40.5° ± 16°gain) and the average external rotation improved from 33.2°to 82.7° (a 49.5° ± 23.9° gain). Although there was a significant difference between Group I and II for preoperative abduction (p = 0.000) and abduction gain in degrees (p = 0.001), the difference between postoperative values of both groups was not significant (p = 0.268). There was also no significant difference between the two groups in the preoperative external rotation, the external rotation gain and the postoperative external rotation (p = 0.163, p = 0.181 and p = 0.803, respectively).

**Conclusions:**

Obstetric palsy patients with shoulder sequela who had a preoperative abduction less than 90°hadas good functional results using latissimus dorsi, teres major muscle transfer and subscapularis muscle release as the patients who hada preoperative abduction greater than 90°.

## Background

Although the extent and severity of the deformity in obstetrical palsy may vary from patient to patient, shoulder is the most frequently affected joint. Many clinical and radiological classification systems had been described to address the problem and tailor the solution at the shoulder [1-5]. Among deformities affecting this joint, internal rotation contracture causing limitation of abduction and external rotation and dislocation of the shoulder is commonly observed on the follow up [4]. The shoulder instability was not caused directly by obstetrical trauma but related to a dynamic phenomenon of muscle imbalance. It was discovered that subscapularis muscle usually recovers more quickly than the external rotators and abductors [6,7]. Despite initial conservative therapy, patients with partial recovery may develop internal rotation and adduction contractures at the shoulder. If this persists long enough, flattening of the humeral head and joint incongruence will ensue. Under these conditions the main goal of the treatment is to reestablish the muscle equilibrium. The first step is to treat the muscle release such as the posterior subscapularis slide or the modified anterior subscapularis tendon lengtening procedures. Afterwards, muscle transfer is performed to restore the missing external rotation and abduction function in patients with congruent glenohumeral joint and limited glenohumeral deformity. However external rotational humeral osteotomy is preferred in patients with similar external rotation weakness, internal rotation contracture but with severe glenohumeral deformity [1].

Usually release and transfer procedures are seperated depending on the age of the patient and condition of the shoulder but in older age group (>4 years), it is preferred to combine the shoulder release and muscle transfer simultaneously [6-8].

Some authors perform other muscle transfers like levator scapulae and trapezius along with latissimus dorsi and teres major only for total flail shoulders [9], others claim that if the preoperative abduction degree of the shoulder is less than 90° indicating a week deltoid and external rotator muscles, a trapezius muscle transfer must be added to latissimus dorsi and teres major transfers to achieve significant improvement of both abduction and external rotation [10].

In this study, we compared the results of combined release and tendon transfer operations performed at the same stage because of the late presentation of the cases, for shoulder abduction and external rotation in two groups of patients who did not have any surgical treatment before. In one group preoperative abduction degree was less than 90°, while in the other group it was equal or more than 90°. We compared the gains of abduction and external rotation in two groups and evaluated whether the preoperative abduction degree affects the functional outcome and in patients with preoperative abduction degree <90° and also another muscle transfer is needed along with latissimus dorsi and teres major transfers.

## Methods

### General data about the patients

Between 1998 and 2002, 46 children with obstetrical brachial plexus palsy who had no surgical treatment before (30 male and 16 female) were operated to restore shoulder abduction and external rotation in our clinic. The average age at surgery was 7.6 years (3–16). The patients had an average follow up of 40.8 months (range 24 to 60 months).

Involvement of right side was seen in 30 patients and the left side in 16 patients. No patient had bilateral involvement. All of the patients were vaginally delivered with vertex presentations. Obstetrical history revealed that most mothers were multiparous and 8 of the patients were delivered with the help of forceps or vacuum. The mean birth weight of the patients was 4.5 kg (3–6.6 kg).

The pool of the patients consisted 12 of patients with C5–C6 spinal root involvement, while 11 of the patients had additional C7 involvement. Finally 23 of the patients had total brachial plexus roots involvement. Accompanying birth complications were fracture of clavicle in one case, injury of sternocleidomastoideus muscle in one case and Horner's Syndrome in 2 cases.

Table 1 [see Additional file 1] summarizes the specific qualifications, preoperative and postoperative evaluation values of all patients.

We compared the postoperative results of the patients who had preoperative abduction less than 90° [Group I: n = 37, mean abduction 62.5° (20°–85°) and mean external rotation 21.4° (0–80°)] with the patients who had preoperative abduction equal or more than 90° [Group II: n = 9, mean abduction 99.4° (90–110) and mean external rotation 33.2° (0–65)], in terms of abduction and external rotation function with angle measurements and Mallet classification. The statistical analysis was performed with analysis of variance (ANOVA) and Student's t-test. Statistical significance was presumed at *p *< 0.05.

### Preoperative and postoperative evaluation

**Figure 1:**
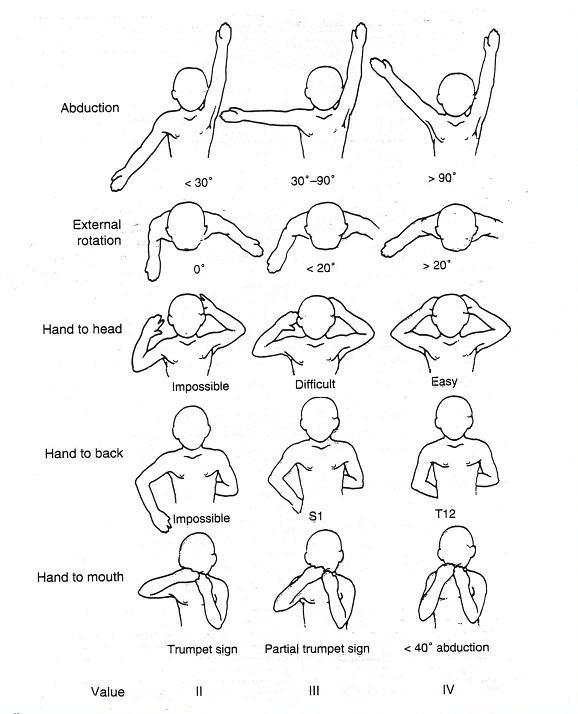
Mallet classification. Grade 0 indicates no movement in the desired plane, Grade V is full movement.

Preoperative and postoperative active and passive range of motion degrees of abduction and external rotation were measured, videos were recorded during shoulder abduction and external rotation and Mallet scores (Figure 1) were noted. Abduction degree is measured at standing position and external rotation is measured in prone position with 90° shoulder abduction and at 90° elbow flexion.

Radiography of the shoulder in adduction and 90° abduction and axial magnetic resonance imaging of the shoulder was performed. Shoulder deformity was classified according to Waters-Peljovich grading system, Table 2, and the patients with type I and type II deformities were included in the series [1].

Most of the times, preoperative physical examination revealed weakness of the deltoid muscle and external rotators as well as co-contraction of pectoralis major, latissimus dorsi and teres major muscles at the anterior and posterior margin of the axillary fossa during shoulder elevation specially in Group I patients.

### Operative technique

**Figure 2:**
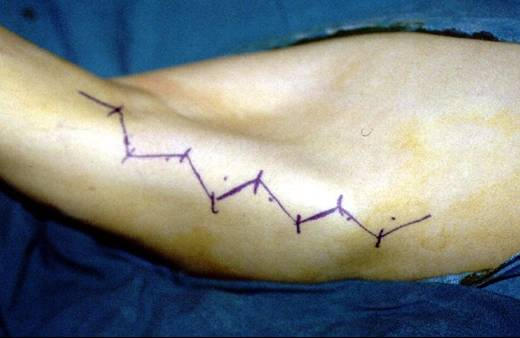
Operative incision. Posterior zigzag incision parallel to the lateral border of scapula is planned for preparing the conjoined tendon of latissimus dorsi and teres major.

**Figure 3:**
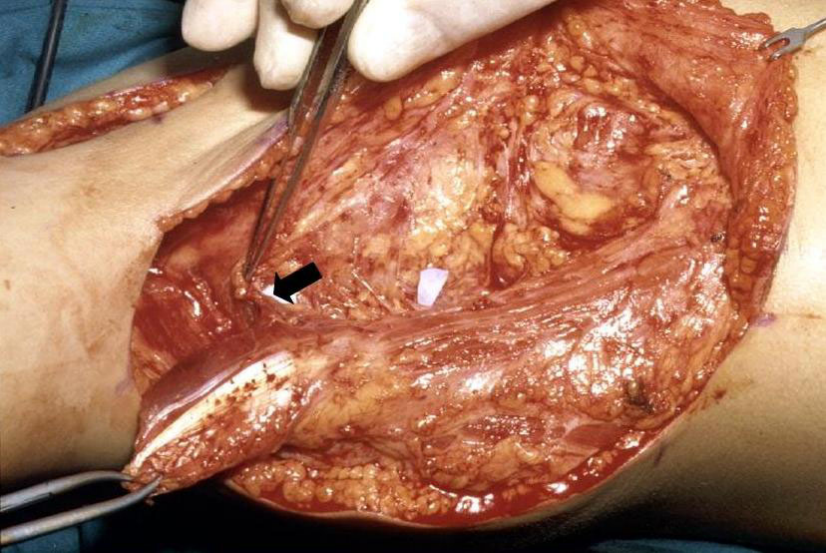
Conjoined tendon preperation. The conjoined tendon is ready for the transfer and its pedicle is pointed medial side of the muscles. Arrow indicates muscle pedicle.

**Figure 4:**
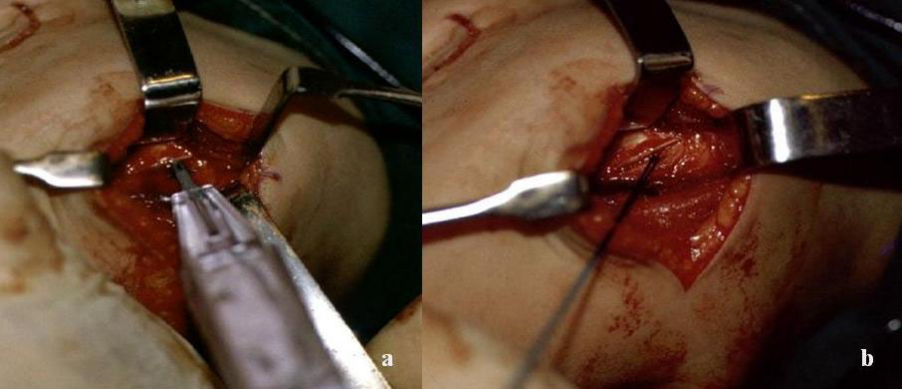
Rigid fixation. Rotator Cuff Quick Anchor which had size 2 with green ethibond polyster sutures on it, was applied at the insertion point of infraspinatus muscle.

**Figure 5:**
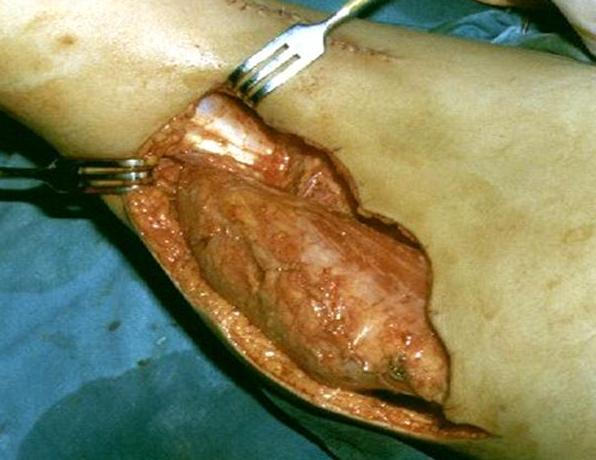
Tendon reinsertion. The conjoined tendon was transferred to the posterior deltoid incision and reinsertion of the conjoined tendon to humerus was achieved while the arm was at 90° abduction and full external rotation.

**Figure 6:**
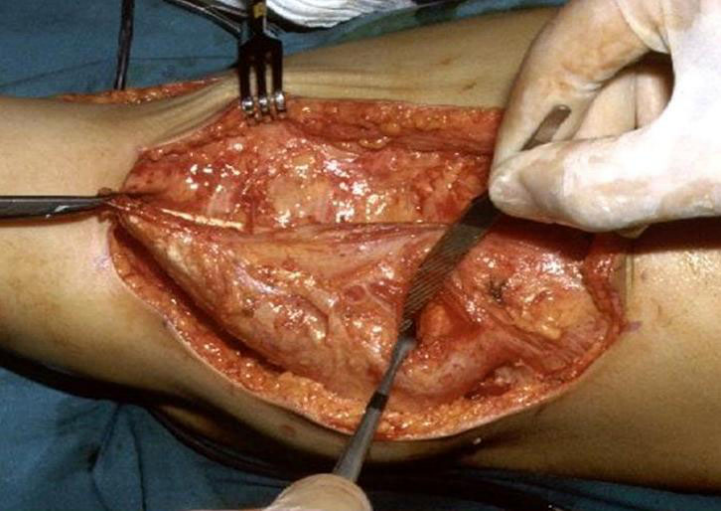
Subscapular muscle release. Subscapularis muscle is released from the anterior surface of the scapula subperiostally.

In our series we used a technique similar to the Hoffer technique [8]. We observed tightness and/or hypertrophy of latissimus dorsi, teres major, subscapularis and sometimes pectoralis major muscles intraoperatively. We performed subscapularis muscle release and the latissimus dorsi and teres major muscle transfer at the same session since the mean age at surgery was 7.6 years. Patients were placed in the lateral decubitus position and conjoined tendon of latissimus dorsi and teres major was explored with a posterior zigzag incision parallel to the lateral border of scapula to prevent scar contracture (Figure 2). Extensive dissection of the conjoined tendon and the related muscles from the surrounding structures was needed so that the conjoined tendon could reach to its new insertion easily. While preparing the muscles for transfer care must be taken not to injure the pedicles, which were located at medial side of these muscles (Figure 3). The next step was detachment of the conjoined tendon from its insertion on the inner side of the humerus by retracting the neurovascular structures of the arm superiorly. Afterwards a tunnel was prepared between long head of triceps and deltoid muscle with an extensive care to prevent injury to the axillary nerve. Rotator Cuff Quick Anchor^® ^Plus (Johnson & Johnson) which had size 2 green ethibond polyster sutures on it, was applied at the insertion point of infraspinatus muscle on the rotator cuff (Figure 4). The suture material was transferred to the posterior incision from the tunnel and the conjoint tendon was interwoven with this suture material. Then conjoined tendon was transferred to the posterior deltoid incision. Finally, reinsertion of the conjoined tendon to humerus was achieved while the arm was at 90° abduction and full external rotation (Figure 5). Fractional tenotomy or Z plasty procedure was applied to the pectoralis major tendon, if necessary, through an anterior axillary incision or by retracting posterior incision superiorly. Subscapularis muscle was released from the anterior surface of the scapula subperiostally from a small incision at the lateral side of the scapula carefuly, in order to prevent injury to the pedicle of lattisimus dorsi and teres major muscles (Figure 6). A cast, stabilizing the shoulder at 90° abduction and 90° external rotation and elbow at 90° flexion was applied for five weeks and physiotherapy was started after the removal of the cast under the control of custom-made splint.

## Results

The average abduction degree improved from 62.5° (20°–85°) to 131.4° (90°–165°) and the average external rotation degree improved from 21.4° (0°–80°) to 82.6° (30°–95°) in Group I. We obtained 68.9° ± 22.9° (109%) gain for abduction and 61.1° ± 23° (285%) gain for external rotation. The difference between preoperative and postoperative values of abduction and external rotation was significant (ANOVA, F = 265 p = 0.000, F = 201 p = 0.000, respectively).

The average abduction degree improved from 99.4° (90°–110°) to 140° (110°–170°) and the average external rotation degree improved from 33.2° (0°–65°) to 82.7° (45°–90°) in Group II. We obtained 40.5° ± 16° (40%) gain for abduction and 49.5° ± 23.9° (149%) gain for external rotation. The difference between preoperative and postoperative values of abduction and external rotation was significant (ANOVA, F = 25 p = 0.000 and F = 32.3 p = 0.000, respectively). The results were summarized in Table 3.

Although difference between preoperative abduction and abduction gain values in terms of degrees of Group I and Group II were significant (ANOVA, F= 43.1 p = 0.000 and F = 12 p = 0.001, respectively), the difference between postoperative values of both groups was insignificant (ANOVA, F = 1.257 p = 0.268).

There was also no significant difference between the preoperative external rotation, the external rotation gain and the postoperative external rotation for both groups (ANOVA, F = 2.017 p = 0.163, F = 1.848 p = 0.181 and F = 0.063 p = 0.803, respectively).

**Figure 7:**
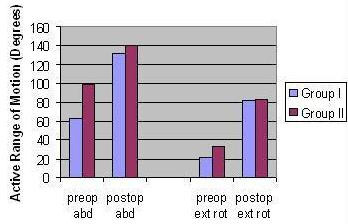
Preoperative and postoperative abduction (abd) and external rotation (ext rot) range of motion degrees of Group I and Group II patients.

The results of both groups were shown in graphics in Figure 7.

Mean Mallet scores increased from 2.8 to 3.9 for global abduction, from 2.5 to 3.9 for global external rotation, from 2.1 to 3.6 for hand to head and from 2.5 to 3.5 for hand to mouth for Group I. Hand to back Mallet score decreased from 2.5 to 2.2. All the differences between preoperative and postoperative values were found significant according to the Student's t-test (t = -17.14 p = 0.000 for abduction score, t = -12.04 p = 0.000 for external rotation score, t = -13.06 p = 0.000 for hand to head score, t = 2.372 p = 0.023 for hand to back score and t = -7.361 p = 0.000 for hand to mouth score).

Mean Mallet scores increased from 3.5 to 4 for global abduction, from 2.8 to 4 for global external rotation, from 2.7 to 4 for hand to head and from 3.2 to 3.5 for hand to mouth for Group II. Hand to back Mallet score decreased from 2.8 to 2.1. All the differences, apart from hand to mouth score, between preoperative and postoperative values were found significant according to the Student's t-test (t = -2.53 p = 0.035 for abduction score, t = -3.592 p = 0.007 for external rotation score, t = -4.4 p = 0.002 for hand to head score and t = 2.8 p = 0.023 for hand to back score). The difference between preoperative and postoperative hand to mouth scores was found insignificant (t = -2, p = 0.081).

While the difference between preoperative Mallet scores of Group I and Group II concerning abduction, hand to head and hand to mouth was significant (ANOVA, F = 23.211 p = 0.000, F = 11.407 p = 0.002 and F = 7.692 p = 0.008, respectively), the difference was insignificant for external rotation and hand to back scores (ANOVA, F = 1.393 p = 0.244 and F = 2.475 p = 0.123, respectively). The difference between postoperative values of both groups was insignificant (ANOVA, F = 0.239 p = 0.627 for abduction, F = 0.76 p = 0.388 for external rotation, F = 2.764 p = 0.106 for hand to head, F = 0.354 p = 0.555 for hand to back and F = 0.363 p = 0.555 for hand to mouth).

The results were summarized in Table 4.

**Figure 8:**
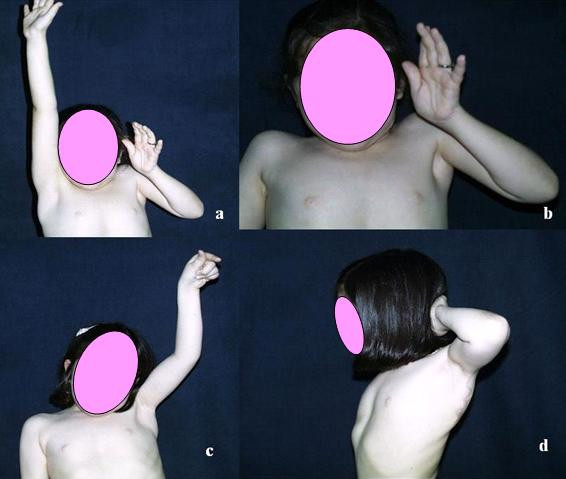
An example case from Group I, Case 6 in Table 1: 7 years old girl with left brachial plexus palsy. (a), (b) Anterior preoperative views of the patient while performing abduction and external rotation of her left arm reveals co-contraction of pectoralis major, latissimus dorsi and teres major muscles at the anterior and posterior margin of the axillary fossa. Preoperative abduction degree was 60° and external rotation degree was 45°. (c) Anterior postoperative view of the patient while performing abduction. (d) Lateral postoperative view of the patient while placing her hand at the nape of her neck. Postoperative abduction degree is 135° and external rotation degree is 87°.

**Figure 9:**
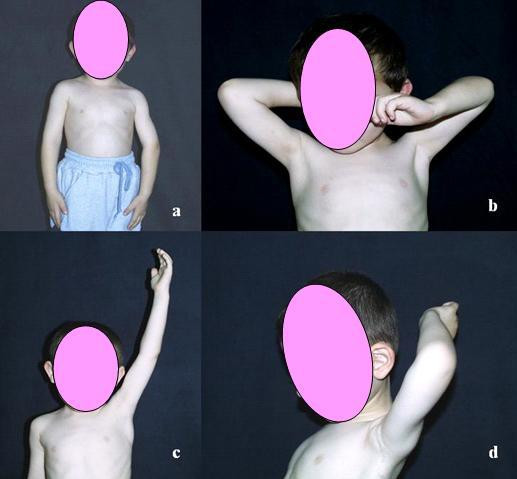
An example case from Group II, Case 38 in Table 1: 5 years old boy with left brachial plexus palsy. (a), (b) Anterior views of the patient while performing abduction of his both arms. Preoperative abduction degree is 110° and external rotation degree is 24°. (c) Anterior postoperative view of the patient while performing abduction. (d) Lateral postoperative view of the patient while placing his hand at the nape of his neck. Postoperative abduction degree is 170° and external rotation degree is 90°.

Example cases from each group can be seen in Figure 8 and 9.

## Discussion

Internal rotation contracture is the most frequent and important secondary deformity of the shoulder in birth palsy. The problem is sometimes addressed by muscle release procedures such as the posterior subscapular slide or an anterior subscapularis tendon lenghtening operations. Once passive external rotation is improved, the child is later assessed for muscle transfers to reconstruct active external rotation if necessary [4].

According to Chang et al [5] there are two types of residual muscle impairment after recovery in the late obstetric brachial plexus palsy: motor recovery with cross-innervation and paralysis or paresis. Contractures of the pectoralis major, teres major, brachialis and biceps muscles, which are most frequently observed, cause the deformity of the shoulder and elbow. The reconstructive strategy include releasing of the antagonistic muscles (elongation of the pectoralis major and latissimus dorsi muscles) and augmentation of the paretic muscles (teres major transfer to the infraspinatus muscle for augmentation of shoulder external rotation and abduction and reinsertions of both ends of the clavicular part of the pectoralis major laterally for deltoid augmentation).

However, there are still many controversies concerning donor muscle choice for transfer, timing and operative tecniques of palliative surgical theraphy for the shoulder deformity.

The infraspinatus muscle works to center the humeral head in the glenoid throughout elevation. External rotation of the shoulder allows greater arm elevation by clearing the greater tuberosity from impingement by the coracoacromial arch. External rotation of the humerus also positions the long head biceps centrally to aid in its function as humeral stabilizer and loosens the inferior glenohumeral ligaments, thereby allowing greater arm elevation. Hence the infraspinatus muscle plays a key role in shoulder elevation as a humeral head stabilizer, an active abductor, and an external rotator of the shoulder [6].

The importance of transferring the teres major and latissimus dorsi as one conjoined tendon and anchoring into the posterior aspect of the greater tuberosity at the insertion of the infraspinatus similar to Hoffer method is augmentation of the weakened infraspinatus. Transfer with this technique instead of rerouting around humeral neck enables a stronger external rotator power because of the increased mechanical advantage at its insertion in the humeral head as opposed to the humeral shaft. The reason for the dramatic improvement of shoulder abduction after latissimus muscle transfer is probably because the transfer enhances the stabilizing effect of the rotator cuff which enables the deltoid to act more effectively, this phenomenon was called "force couple" effect by Phipps and Hoffer [11].

In many centers, muscle release procedures are performed before the age of two years, however for older children tendon transfer to restore abduction and external rotation is added [12]. It is accepted that the corrective procedures to rebuild the muscle equilibrium are best undertaken before permanent bony deformity occurs at 3 to 4 years of age [13].

Gilbert [14] suggested that release of the subscapularis is indicated if the external rotation does not improve more than 20°. Based on his 5 years of follow up, he reported excellent results after subscapularis release especially in patients before the age of 2 years. Raimondi also waits for the active external rotation due to the reinforcement of weak external rotator muscles after subscapular muscle release procedure in early ages but since recovery of the external rotators cannot be expected, he preferres the tendon transfer and muscle release operations at the same time in children older than 4 years of age [9].

Muhlig et al [12] described a common policy accepted by most of the centers. According to this; if passive external rotation of the shoulder stays < 30°, surgical treatment is indicated. If there is no posterior displacement of the humeral head than a subscapular slide will be used. However, if there is posterior displacement of the humeral head than subscapular lengthening by an anterior approach will be preferred. Indications for tendon transfer for improving external rotation and abduction are determined as well. If infraspinatus muscle does not show signs of reinnervation by the age of 2 years, a muscle transfer should be added to the subscapularis lengthening to avoid recurrence. If there is a fixed medial rotation contracture and posterior luxation of the humeral head with deformities of the glenoid than derotational osteotomy of the humerus should be added to the subscapularis lengthening.

As all of our patients were older than 2 years of age, we performed latissimus dorsi and teres major transfer at the same session with subscapularis and pectoralis major release.

In total flail shoulders, despite a certain degree of innervation, the functional results of shoulder corresponds to zero with the absence of a strong latissimus dorsi. In that condition, the levator scapulae muscle is utilised as an intrinsic stabilizer of glenohumeral joint and trapezius muscle is used as a prime mover for shoulder abduction with or without latissimus dorsi and teres major transfers [9].

Gilbert in his series of 44 patients with transfer of latissimus dorsi, the improvement of abduction was satisfactory in the shoulders which preoperatively coded as Grade III (Shoulder abduction is between 90°–120°, external rotation is between 0°–30°) or more, but not in those coded as Grade II (Shoulder abduction is between 45°–90°, external rotation is to neutral) or less. Hence he thought it may be necessary to add a concomitant transfer of the trapezius to the patients whose abduction of the shoulder was weak or absent [15].

Chen et al [10] asserted the need for an additional trapezius muscle transfer for shoulder of the patients who had less than 90° abduction to increase the success of the classic latissimus dorsi + teres major transfer. They transferred latissimus dorsi by fixing its tendon to the insertion of the infraspinatus and tenotomized the teres major and then attached to the belly of the latissimus dorsi and found out in their early stage of treatment that, 10 of 18 cases with abduction less than 90°, with transfer of the latissimus dorsi and the teres major, patients gained no improvement of abduction but some recovery of external rotation, while five of seven patients with abduction equal or more than 90°, made significant progress in both abduction and external rotation.

Al-Qattan [16] performed latissimus dorsi transfer on 12 children with variable preoperative shoulder abduction (range 60–150°, mean 100°) and postoperatively ten children achieved a modified Mallet score of 4 and were able to reach the occiput easily and they had mean 140° active shoulder abduction (range 90–170°). Hence the author also did not find any difference in patients with weak or strong preoperative abduction.

It is our opinion that in our Group I patients, the co-contraction between shoulder abductors (supraspinatus, infraspinatus and deltoid) and adductors (mainly, pectoralis major, teres major and latissimus dorsi) and also subscapularis muscle tightness cause limitation of shoulder elevation. If antagonistic muscles (teres major and latissimus dorsi) are transferred for the paretic muscles (infraspinatus) and the pectoralis major and subscapularis muscles are released with preserving their shoulder stability function, these children can have as succesfull postoperative shoulder abduction and external rotation as the children in Group II, who has less cross innervation hence better preoperative abduction value. Group I and II patient had almost similar postoperative mean abduction (131.4° & 140°, respectively) and external rotation values (82.6° & 82.7°, respectively).

Extensive dissection of latissimus dorsi and teres major muscles from the surrounding structures gave us the opportunity to utilize both muscles for transfer, without any difficulty during the passage of the conjoined tendon through the tunnel which was prepared between long head of triceps and deltoid muscle, and also during reinsertion to the humerus.

Several authors reported recurrences of the deformity in terms of reduction of external rotation and abduction gain. Two of the 12 children in Al-Qattan series [16] and three of 35 cases in Phipps and Hoffer series [11] had recurrence of the deformity. Al-Qattan [16] classified the possible cause of this late complication as recurrence of the internal rotation contracture (mainly in the subscapularis), gradual contracture of the teres major as part of the inferior glenohumeral angle contracture and co-contraction of the muscles. We did not have any recurrence of the deformity during the follow-up period which may be related to the use of rigid fixation with bone anchors for reinsertion of the conjoint tendon.

We believe that in cases who has congruent glenohumeral joint (Type I-III Waters-Peljovich grading system), and deltoid muscle strength of M3–M4 (British Medical Research Council evaluation) but weak or absent external rotation, if the latissimus dorsi and teres major muscles have sufficient strength (M3 or more), the ideal procedure is transfer of latissimus dorsi and /or teres major onto the posterior aspect of the greater tuberosity of humerus, at the insertion of the infraspinatus. So we are not totally convinced about adding trapezius muscle transfer concomitantly with the latissimus dorsi + teres major transfer session. We rather preserve this muscle for the patients that could not achieve enough shoulder abduction after the first operation.

## Conclusions

Since our study was not randomised to treatment, the groups did not comprise equal number of patients, the assessments were not performed by independent observers but by a physiotherapy group of our team at postoperative 24 – 60 months (not at the same time for everyone), we would like to interpret our conclusions cautiously.

Almost near normal shoulder function can still be reached in children who could not receive primary early neural reconstruction, by combined muscle release and muscle transfer operations which are performed before the severe glenohumeral deformities occur.

We found out that the patients with obstetric palsy shoulder sequela who had a preoperative abduction value less than 90° could have good functional results as the patients who had preoperative abduction values equal or more than 90°, with latissimus dorsi, teres major muscle transfer and subscapularis muscle release.

## Competing interests

None declared.

## Authors' contributions

All authors participated in the design of the study, operations and drafting the manuscript. All authors read and approved the final manuscript.

## Pre-publication history

The pre-publication history for this paper can be accessed here:



## Supplementary Material

Additional File 1 Preoperative and postoperative range motion degrees and Mallet scores of the patients. Patients with bold numbers are in Group II with preoperative abduction values ≥ 90° and the others are in Group I with preoperative abduction values < 90° (Abd Deg: abduction degree, Ex. Rot. Deg: external rotation degree). Passive range of motion degrees are in parenthesis. Click here for file

## Figures and Tables

**Table 2:** Radiographic classification of glenohumeral deformity (Waters and Peljovich).

**Type I**	Normal glenoid (<5° retroversion difference versus contralateral normal)
**Type II**	Minimal deformity (>5° retroversion difference)
**Type III**	Moderate deformity (posterior humeral head subluxation)
**Type IV**	Severe deformity (posterior humeral head subluxation with false glenoid)
**Type V**	Severe flattening of humeral head and glenoid +/- complete dislocation
**Type VI**	Infantile glenohumeral dislocation
**Type VII**	Proximal humeral growth arrest

**Table 3:** Abduction and external rotation evaluation of patients in Group I and Group II.

	Preoperative Abduction	Postoperative Abduction	Abduction Gain	Preoperative External Rotation	Postoperative External Rotation	External Rotation Gain
Group I (n = 37)	62.5°# (20°–85°)	131.4° * (90°–165°)	68.9° ± 22.9 # (109%)	21.4° (0–80°)	82.6° * (30°–95°)	61.1° ± 23° (285%)
Group II (n = 9)	99.4°# (90°–110°)	140° * (110°–170°)	40.5° ± 16°# (49.5%)	33.2° (0–65°)	82.7° * (45°–90°)	49.5° ± 23.9° (149%)

"*" sign represents significant difference between pre and postoperative values in the groups and "#" sign represents significant difference between Group I and Group II.

**Table 4:** Preoperative and postoperative Mallet scoring.

	Mallet Classification									
	
	Abduction		External Rotation		Hand to Head		Hand to Mouth		Hand to Back	
	
	Preop	Postop	Preop	Postop	Preop	Postop	Preop	Postop	Preop	Postop
Group I (n = 37)	2.8#	3.9*	2.5	3.9*	2.1#	3.6*	2.5#	3.5*	2.5	2.2*
Group II (n = 9)	3.5#	4*	2.8	4*	2.7#	4*	3.2#	3.5	2.8	2.1*

"*" sign represents significant difference between pre and postoperative values in the groups and "#" sign represents significant difference between Group I and Group II.
