# Supplementary material for: Does preoperative abduction value affect functional outcome of combined muscle transfer and release procedures in obstetrical palsy patients with shoulder involvement?
Source: BMC Musculoskelet Disord. 2004 Aug 3;5:25. doi: 10.1186/1471-2474-5-25 (PMC514557; doi:10.1186/1471-2474-5-25)
Supplement: Additional File 1 — Preoperative and postoperative range motion degrees and Mallet scores of the patients. Patients with bold numbers are in Group II with preoperative abduction values ≥ 90° and the others are in Group I with preoperative abduction values < 90° (Abd Deg: abduction degree, Ex. Rot. Deg: external rotation degree). Passive range of motion degrees are in parenthesis. [file 1471-2474-5-25-S1.doc]

|  | | | | | Preoperative | | | | | | | Postoperative | | | | | | |
| --- | --- | --- | --- | --- | --- | --- | --- | --- | --- | --- | --- | --- | --- | --- | --- | --- | --- | --- |
| N | Name | Sex  M/F | Age at surgery | Follow up time  (months) | Abd Deg | Ex Rot  Deg | Mallet  Abd | Mallet  Ex Rot | Mallet  Hand to  head | Mallet  Hand to  back | Mallet  Hand to mouth | Abd Deg | Ex  Rot  Deg | Mallet  Abd | Mallet  Ex Rot | Mallet  Hand to  head | Mallet  Hand to  back | Mallet  Hand to mouth |
| 1 | AA | F | 5 | 29 | 60  (180) | 50  (90) | 3 | 3 | 2 | 2 | 3 | 130  (180) | 70  (90) | 4 | 3 | 3 | 2 | 3 |
| 2 | AGe | M | 16 | 41 | 85  (140) | 80  (90) | 3 | 3 | 3 | 3 | 4 | 100  (140) | 95  (95) | 4 | 4 | 4 | 2 | 3 |
| 3 | AM | M | 5 | 30 | 85  (180) | 10  (90) | 3 | 2 | 2 | 2 | 2 | 165  (180) | 90  (110) | 4 | 4 | 4 | 2 | 3 |
| 4 | AÜ | M | 16 | 25 | 70  (140) | 60  (95) | 3 | 3 | 2 | 3 | 3 | 125  (140) | 95  (95) | 4 | 4 | 4 | 2 | 3 |
| 5 | BDa | M | 3 | 30 | 50  (175) | 0  (90) | 3 | 2 | 2 | 2 | 2 | 115  (175) | 85  (90) | 4 | 4 | 3 | 2 | 3 |
| 6 | BDe | F | 7 | 48 | 60  (175) | 45  (90) | 3 | 3 | 3 | 3 | 4 | 135  (180) | 87  (100) | 4 | 4 | 4 | 2 | 4 |
| 7 | BÖ | M | 9 | 50 | 80  (180) | 40  (90) | 3 | 3 | 2 | 2 | 2 | 150  (180) | 90  (90) | 4 | 4 | 4 | 2 | 4 |
| 8 | BS | M | 3 | 39 | 60  (170) | 0  (90) | 3 | 2 | 2 | 3 | 2 | 140  (170) | 85  (90) | 4 | 4 | 4 | 2 | 4 |
| 9 | ÇD | M | 4 | 31 | 75  (175) | 0  (75) | 3 | 2 | 2 | 3 | 2 | 160  (175) | 70  (90) | 4 | 4 | 3 | 2 | 2 |
| 10 | ET | F | 6 | 29 | 75  (180) | 18  (85) | 3 | 2 | 2 | 3 | 2 | 140  (180) | 90  (125) | 4 | 4 | 4 | 2 | 3 |
| 11 | FA | M | 5 | 51 | 75  (170) | 10  (95) | 3 | 3 | 2 | 2 | 2 | 140  (170) | 85  (95) | 4 | 4 | 4 | 2 | 4 |
| 12 | FT | M | 4 | 26 | 75  (180) | 60  (100) | 3 | 3 | 2 | 2 | 3 | 160  (180) | 95  (100) | 4 | 4 | 4 | 2 | 4 |
| 13 | GA | F | 4 | 50 | 35  (140) | 0  (90) | 3 | 2 | 2 | 2 | 2 | 100  (140) | 45  (90) | 4 | 4 | 4 | 2 | 4 |
| 14 | GAk | F | 12 | 24 | 62  (140) | 20  (80) | 3 | 3 | 2 | 4 | 3 | 110  (165) | 70  (90) | 4 | 4 | 4 | 4 | 4 |
| 15 | HK | F | 8 | 45 | 60  (170) | 0  (90) | 2 | 3 | 2 | 2 | 3 | 155  (170) | 90  (90) | 4 | 4 | 4 | 2 | 4 |
| 16 | HT | M | 10 | 50 | 40  (160) | 0  (95) | 3 | 2 | 2 | 3 | 3 | 135  (160) | 75  (85) | 4 | 4 | 2 | 2 | 3 |
| 17 | İBÇ | M | 6 | 30 | 80  (180) | 45  (90) | 3 | 3 | 2 | 3 | 2 | 140  (180) | 85  (90) | 4 | 4 | 4 | 2 | 3 |
| 18 | İCS | M | 5 | 26 | 40  (180) | 5  (90) | 3 | 3 | 2 | 2 | 3 | 95  (180) | 90  (90) | 4 | 4 | 2 | 2 | 3 |
| 19 | İD | M | 5 | 50 | 35  (160) | 50  (90) | 3 | 4 | 2 | 3 | 2 | 155  (160) | 90  (90) | 4 | 4 | 4 | 4 | 4 |
| 20 | KA | M | 9 | 60 | 50  (140) | 0  (85) | 3 | 2 | 2 | 3 | 2 | 120  (140) | 85  (85) | 4 | 4 | 4 | 2 | 3 |
| 21 | KG | F | 3 | 36 | 75  (160) | 20  (90) | 3 | 2 | 2 | 2 | 2 | 145  (160) | 90  (90) | 4 | 4 | 4 | 2 | 4 |
| 22 | KGü | M | 7 | 35 | 80  (180) | 15  (90) | 3 | 2 | 2 | 3 | 2 | 145  (180) | 90  (90) | 4 | 4 | 4 | 2 | 3 |
| 23 | KŞ | M | 9 | 59 | 40  (120) | 0  (80) | 2 | 3 | 2 | 2 | 3 | 105  (120) | 80  (80) | 4 | 4 | 3 | 2 | 4 |
| 24 | MG | M | 8 | 50 | 80  (140) | 10  (90) | 3 | 2 | 2 | 2 | 2 | 120  (140) | 80  (90) | 4 | 4 | 4 | 2 | 3 |
| 25 | MP | F | 7 | 45 | 55  (160) | 23  (95) | 2 | 2 | 2 | 3 | 2 | 140  (180) | 90  (90) | 4 | 4 | 4 | 3 | 4 |
| 26 | MY | F | 8 | 50 | 65  (140) | 45  (90) | 3 | 4 | 2 | 3 | 2 | 120  (140) | 90  (90) | 4 | 4 | 4 | 2 | 3 |
| 27 | NA | F | 13 | 35 | 60  (145) | 0  (85) | 2 | 2 | 2 | 3 | 3 | 120  (150) | 80  (95) | 4 | 3 | 4 | 2 | 4 |
| 28 | NE | F | 13 | 36 | 67  (160) | 0  (61) | 3 | 2 | 2 | 2 | 2 | 120  (!60) | 70  (110) | 4 | 4 | 4 | 2 | 4 |
| 29 | NT | F | 9 | 28 | 65  (160) | 20  (90) | 3 | 2 | 2 | 3 | 2 | 145  (160) | 90  (90) | 4 | 4 | 3 | 2 | 3 |
| 30 | ÖB | M | 9 | 60 | 65  (140) | 30  (90) | 3 | 3 | 2 | 3 | 2 | 130  (140) | 80  (90) | 4 | 4 | 4 | 4 | 3 |
| 31 | RY | M | 4 | 60 | 60  (150) | 0  (60) | 3 | 2 | 2 | 2 | 2 | 120  (170) | 85  (120) | 4 | 4 | 4 | 2 | 3 |
| 32 | SA | M | 5 | 59 | 20  (140) | 0  (90) | 2 | 3 | 3 | 2 | 3 | 130  (160) | 95  (90) | 4 | 4 | 4 | 4 | 4 |
| 33 | SHU | F | 9 | 28 | 75  (170) | 55  (90) | 3 | 3 | 3 | 2 | 3 | 164  (180) | 90  (90) | 4 | 4 | 4 | 2 | 4 |
| 34 | SY | M | 16 | 24 | 40  (170) | 20  (90) | 3 | 3 | 2 | 2 | 2 | 150  (170) | 30  (70) | 4 | 3 | 2 | 2 | 3 |
| 35 | TT | M | 10 | 38 | 75  (180) | 43  (85) | 3 | 3 | 3 | 2 | 4 | 110  (155) | 80  (90) | 4 | 4 | 4 | 2 | 4 |
| 36 | UÇ | F | 8 | 50 | 60  (160) | 20  (80) | 3 | 3 | 2 | 2 | 3 | 140  (160) | 80  (80) | 4 | 4 | 3 | 2 | 3 |
| 37 | ZG | F | 16 | 50 | 80  (100) | 0  (90) | 3 | 2 | 3 | 3 | 3 | 90  (100) | 90  (90) | 3 | 4 | 3 | 2 | 3 |
| **38** | BY | M | 5 | 38 | 110  (180) | 24  (85) | 4 | 2 | 3 | 4 | 4 | 170  (180) | 90  (90) | 4 | 4 | 4 | 2 | 4 |
| **39** | EC | F | 5 | 50 | 90  (180) | 10  (85) | 3 | 2 | 2 | 2 | 3 | 120  (180) | 80  (85) | 4 | 4 | 4 | 2 | 3 |
| **40** | EK | M | 7 | 50 | 90  (170) | 20  (80) | 3 | 3 | 2 | 2 | 2 | 110  (170) | 45  (80) | 4 | 4 | 4 | 2 | 3 |
| **41** | RD | M | 9 | 50 | 110  (160) | 65  (90) | 4 | 4 | 4 | 3 | 4 | 145  (180) | 90  (90) | 4 | 4 | 4 | 2 | 4 |
| **42** | RFD | M | 6 | 36 | 95  (180) | 0  (90) | 4 | 2 | 2 | 3 | 2 | 145  (180) | 90  (90) | 4 | 4 | 4 | 2 | 3 |
| **43** | SÇ | M | 7 | 30 | 90  (180) | 55  (90) | 3 | 4 | 3 | 2 | 3 | 110  (180) | 90  (90) | 4 | 4 | 4 | 2 | 3 |
| **44** | ÜGB | F | 4 | 28 | 105  (180) | 30  (90) | 4 | 3 | 3 | 4 | 4 | 170  (180) | 90  (90) | 4 | 4 | 4 | 2 | 4 |
| **45** | YT | M | 4 | 50 | 100  (155) | 55  (90) | 3 | 2 | 2 | 2 | 3 | 145  (180) | 90  (90) | 4 | 4 | 4 | 2 | 4 |
| **46** | İDo | M | 7 | 38 | 105  (150) | 40  (90) | 4 | 4 | 4 | 4 | 4 | 145  (160) | 80  (100) | 4 | 4 | 4 | 3 | 4 |

**Table 1:** Preoperative and postoperative range motion degrees and Mallet scores of the patients. Patients with bold numbers are in Group II with preoperative abduction values ≥ 90º and the others are in Group I with preoperative abduction values < 90º. (Abd Deg: abduction degree, Ex. Rot. Deg: external rotation degree.) Passive range of motion degrees are in parenthesis.
